# Long-term monitoring dataset of fish assemblages impinged at nuclear power plants in northern Taiwan

**DOI:** 10.1038/sdata.2015.71

**Published:** 2015-12-08

**Authors:** Hungyen Chen, Yun-Chih Liao, Ching-Yi Chen, Jeng-I Tsai, Lee-Sea Chen, Kwang-Tsao Shao

**Affiliations:** 1 National Research Institute of Fisheries Science, Fisheries Research Agency, Kanagawa 236-8648, Japan; 2 National Museum of Marine Science and Technology, Keelung 20248, Taiwan, R.O.C; 3 Biodiversity Research Center, Academia Sinica, Taipei 11529, Taiwan, R.O.C

**Keywords:** Biooceanography, Marine biology, Biodiversity, Community ecology

## Abstract

The long-term species diversity patterns in marine fish communities are garnering increasing attention from ecologists and conservation biologists. However, current databases on quantitative abundance information lack consistent long-term time series, which are particularly important in exploring the possible underlying mechanism of community changes and evaluating the effectiveness of biodiversity conservation measures. Here we describe an impinged fish assemblage dataset containing 1, 283, 707 individuals from 439 taxa. Once a month over 19 years (1987–1990 and 2000–2014), we systematically collected the fish killed by impingement upon cooling water intake screens at two nuclear power plants on the northern coast of Taiwan. Because impingement surveys have low sampling errors and can be carried out over many years, they serve as an ideal sampling tool for monitoring how fish diversity and community structure vary over an extended period of time.

## Background & Summary

Monitoring long-term species diversity patterns in marine fish communities is receiving increasing attention from ecologists and other researchers^1–3^. There are extraordinarily large online databases for studying marine biodiversity conservation and sustainable fishery management. However, long-term datasets with consistent sampling method are rather rare, especially the ones of non-reef or non-economical coastal fishes in tropical or subtropical Indo-West Pacific region.

Although Taiwan launched the ‘Environmental Impact Assessment Law’ only in 1994, all its nuclear power plants are required to have baseline data before and after their construction and operation prior to 1994. The construction of the 1st Nuclear Power Plant at Shihmen began in 1971 and its two generators started to operate in 1978 and 1979, respectively. The construction of the 2nd Nuclear Power Plant at Yehliu began in 1974 and its two generators started to operate in 1981 and 1983, respectively. Since 1987, the impingement investigation has been carried out monthly in addition to underwater census to monitor reef fish assemblage and gill-net fishing samples to monitor fishery economics. The original purpose of the investigation was to estimate fishery loss due to cooling water intakes which inevitably impinge fish and marine organisms, but the experiment is also an efficient and economical way to study local fish community structure, especially the pelagic or non-reef associated species which are rarely observed by divers and the non-commercial species which are usually not targeted by traditional fishing methods.

The impingement of fish by power plant cooling water intakes in rivers, lakes, estuaries or coasts provides valuable time-series data for studying aquatic communities^4–9^. The intake screens are ideal location and tool to collect impinged fish: impingement surveys not only have low sampling errors but also can be carried out over a long period of time. Even though the selectivity of the filtering screen may limit the size of fish collected by power plant impingement, it should not detract from the usefulness of impingement data giving the consistency and efficiency of collecting schemes combined with long-term sampling efforts.

Due to complicated seafloor topography, geology and substratum type in the surrounding offshore areas, the seas surrounding Taiwan support a rich and highly diverse aquatic wildlife. The west coast of Taiwan is separated from China by the relatively shallow water of the Taiwan Strait, but the east coast lies on the deep drop-off which leads to the Philippine Sea. The warm Kuroshio Current, flowing along the east coast and Asian continental shelf, is an important source for fisheries. As a result of these factors, studies of fish communities adjacent to Taiwan can contribute to global fish diversity indices. As marine fish diversity has been in serious decline for the past 30–40 years due to human impact of overfishing, pollution and habitat destruction^10^, the fish impingement data provide a valuable resource for elucidating long-term trends in fish community ecology in this area. Moreover, with tractable statistical analysis, the data will help identify key driving forces responsible for the observed temporal changes in fish assemblages in this important fishing ground.

Here we describe the long-term time series dataset of fish killed by impingement at two nuclear power plants on the northern coast of Taiwan. These data have been used to study the temporal and spatial variation of fish community structures^11,12^. For example, the species composition in northern Taiwan was found to be very different from that of southern Taiwan^11^. Liao *et al.* ^13^ reported that the most dominant fish at the 2nd Nuclear Power Plant has changed from an economic species, mullet (*Mugil cephalus*), to lower economic species such as rabbitfish (*Siganus fuscescens*) and spiny puffer (*Diodon holocanthus*), reflecting the decline in fishery resources due to overfishing. Chen *et al.* ^9^ used these data as an example to evaluate a newly proposed index of phylogenetic community diversity and describe the changed phylogenetic species composition. These data can also be used by fishery biologists and ecologists who are interested in depicting and understanding the seasonal pattern of species abundance and composition in relation to anthropogenic pressures, environmental factors, climate change, and trophic interactions. Additionally, because these data are collected monthly, they can be used to assess the effect of different sampling scale in community for ecological monitoring.

## Methods

**Figure 1:**
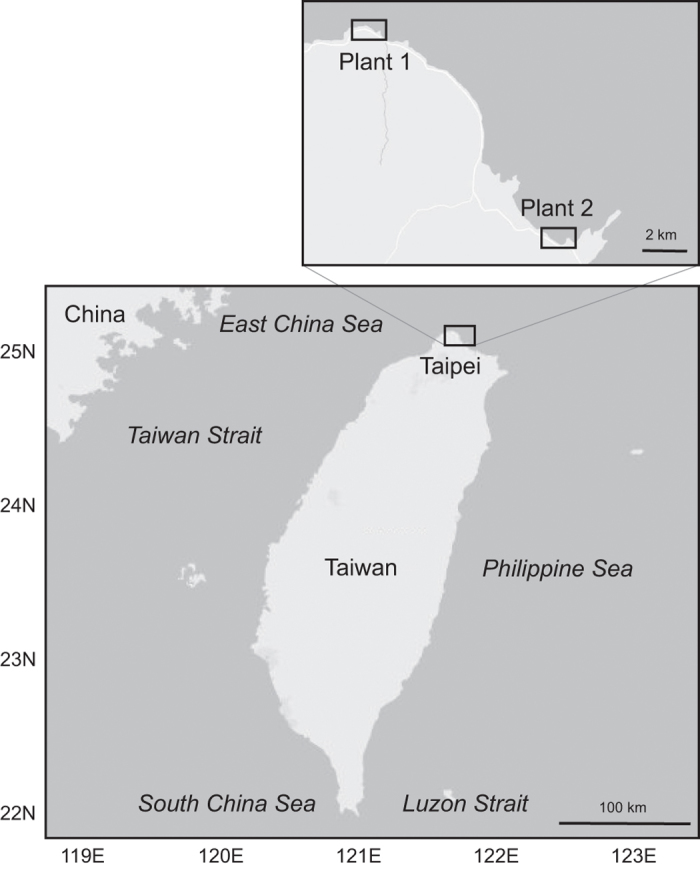
Map of sampling localities: 1st and 2nd Nuclear Power Plants.

The impinged fish community data were collected from the 1st Nuclear Power Plant at Shihmen (25° 17′ 9″ N, 121° 35′ 10″ E) and the 2nd Nuclear Power Plant at Yehliu (25° 12′ 10″ N, 121° 39′ 45″ E) which is about 17 km apart eastward from the 1st. Both plants are situated on the northern coast of Taiwan (Fig. 1). The average generating capacity and the combined water flow velocity of the first plant is 4,680 J and 69 m^3^ s^−1^, and those of the second plant is 7,056 J and 80 m^3^ s^−1^. There was no change in operation that can result in significant changes in the pumped volume, so the combined water flow was constant over years. However, each generator was shut down for maintenance in turn for about one month during winter to spring seasons, which causes the combined water flow velocity to decrease 50%. The sample was collected in opportune days in order to avoid sampling during the maintenance period. Over 6,000,000 m^3^ of water, taken from the near-shore waters off northeastern Taiwan, was used monthly by these two nuclear power plants.

The intake of the first plant was built on a straighter shoreline than that of the second plant, which is located in an open bay along the Yehliu Cape. The sea floors around both intakes are a mixture of coral reefs, gravel, large boulders and sandy patches. From July 1987 to April 1990 and from September 2000 to December 2014 (except for December in 2006 and 2007 at both plants, and January 2007 at the 1st plant), fish samples were collected monthly from the intake screens at both plants for 24 h (from 9 AM to 9 AM) on the date chosen by a systematic sampling method^14^. The mesh size of the intake screen is 20.32 cm×10.16 cm. An oblique bar screen in front of the spur rack was used to prevent heavy debris such as logs. Debris and fishes that were washed off the traveling screens by operating the rotatory machine only when the transported material had accumulated to a certain amount. The impinged material and fish on the 1 cm×1 cm mesh (fish smaller than this size, i.e., fish eggs and larval fishes, may pass through and become entrained) were flushed into a sluice-way and collected in a trash basket suspended outside the pumping house. The waters was returned to the intake bay through the discharge sluice-way. All the fish collected were brought back to the laboratory for sorting, identification and counting. The fish were identified by Doctor Kwang-Tsao Shao and the senior laboratory members using plenty of handbooks of field guide and identification keys. Sampling method and species identification process was constant over years.

The samples collected up to April 1990 were recorded as presence-absence data only. From September 2000 on, the samples were recorded quantitatively, i.e., the number of fish of each species was recorded. As the geographical features of the two intakes are similar to each other, we may treat the monthly samples from both intakes as two replicates.

## Data Records

The dataset includes 407 impinged fish samples collected monthly. The 68 samples collected prior to1990 were recorded as presence-absence data; meaning that the individual numbers of each species were not recorded. The 339 samples collected after 2000 were recorded quantitatively, i.e., species and individual numbers were recorded. The data are represented as list of species name and their abundance collected at both of the two plants as two replicates in each month. In total, the data contain 439 rows (species) and 412 columns (plant, month, and year). The impinged fish data are provided in CSV. To hamper any bad use of the data, we made two distinguished datasets as presence-absence (1987–1990) and abundance (2000–2014) data separately (Data Citation 1).

## Technical Validation

**Figure 2:**
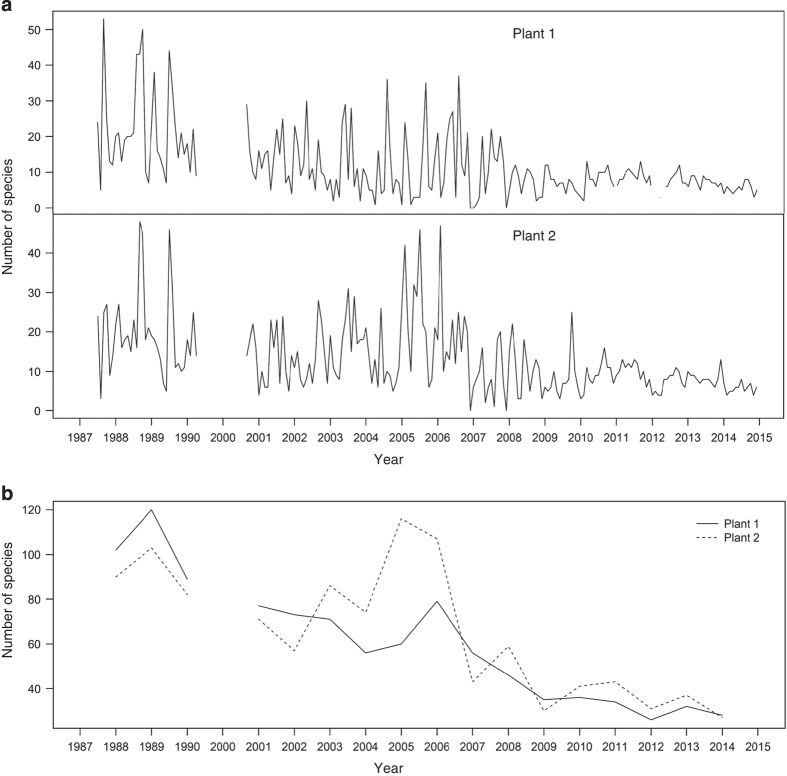
Temporal variation of number of species of impinged fish collected at the 1st and 2nd Nuclear Power Plants in Taiwan during the periods 1987–1990 and 2000–2014. (**a**) Monthly fluctuation. (**b**) Yearly fluctuation.

Four-hundred and thirty-nine species of impinged fish collected at the 1st and 2nd Nuclear Power Plants in Taiwan are recorded in the dataset. Two-hundred and fifty-five species were collected in the 34 months (presence-absence data) from July 1987 to April 1990 and 1, 283, 707 individuals belonging to 335 species were collected in the 170 months from September 2000 to December 2014. The number of species collected per month ranges between 1 and 53 and averages 12.42±9.20 (s.d.). The monthly fluctuations of the number of species are shown in Fig. 2a. These fluctuations became more moderate after 2008, which was due to the disappearance of the seasonal species. The resident fish appeared steadily, but the number of migrant fish declined. Because sampling method, water flow, and species identification process was constant over years, artificial factors should be excluded from the causes of the changed pattern.

**Figure 3:**
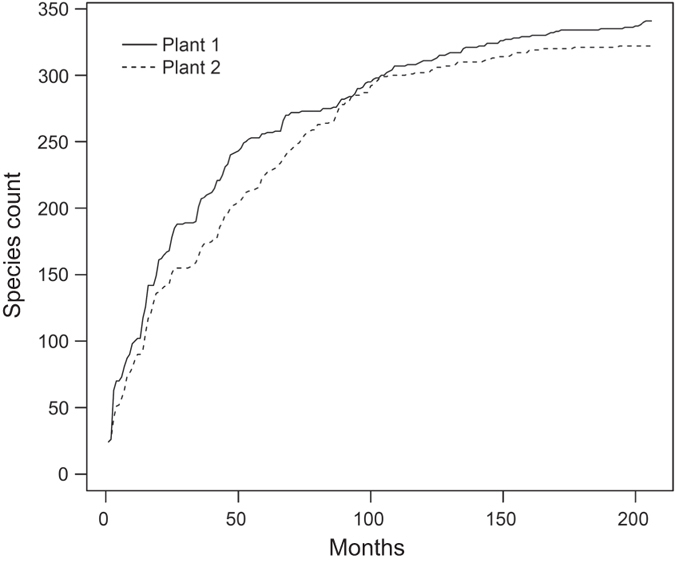
Cumulative curve of species count of impinged fish collected at the 1st and 2nd Nuclear Power Plants in Taiwan during the periods 1987–1990 and 2000–2014 (a total of 206 months).

**Figure 4:**
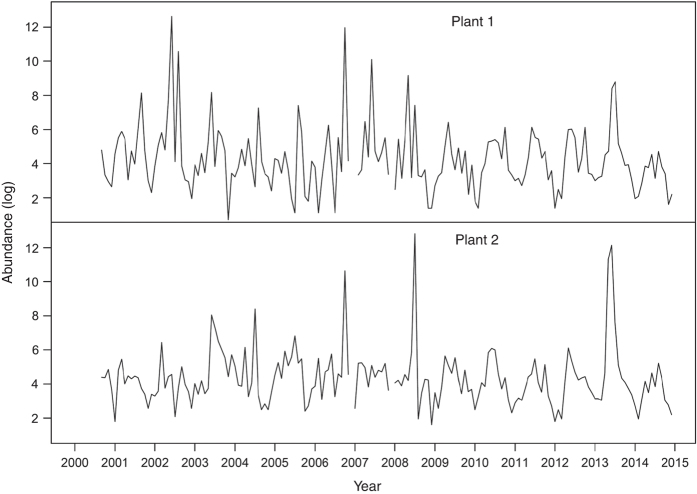
Monthly fluctuations of number of impinged fish collected at the 1st and 2nd Nuclear Power Plants in Taiwan during the period 2000–2014.

Figure 2b shows a decreasing trend of yearly number of species, especially since 2006. The monthly cumulative curve of species count from 1987 to 2014 shows species number may not increase in the further samplings, which suggests that the dataset incorporate a convincing total number of species in the survey area (Fig. 3). The average number of individual for the collections from September 2000 to December 2014 is 3,731.71±29, 298.85 and ranges between 2 and 366,584. Monthly fluctuation of number of individual of impinged fishes collected at the first and second nuclear power plant in Taiwan during the period 2000–2014 is shown in Fig. 4.

The extremely abundant individuals or called ‘massive impingement’ in some specific species were observed in the following months. At the first plant, in June 2002, 305,904 individuals were collected, including *Sardinella gibbosa* (251,300 individuals), *Decapterus maruadsi* (36,750), and *Trachurus japonicus* (13,650); in August 2002, 38, 572 individuals were collected, including *Sardinella gibbosa* (37,810); and in June 2007, 24,196 individuals were collected, including *Siganus fuscescens* (24,146). At the second plant, in July 2008, 366,584 individuals were collected, including *Sardinella lemuru* (358,239); in May 2013, 82,317 individuals were collected, including *Sardinella lemuru* (82,000); and in June 2013, 188,196 individuals were collected, including *Sardinella lemuru* (185,000). These months had no incidence at both plants, besides October 2006. In October 2006, 158,249 and 41, 494 individuals were collected at the first and the second plant. *Encrasicholina punctifer* (158,220 and 41,400) was the most abundant species at both plants. Excluding the above month with the extremely abundant individuals, the average number of individual for the collections becomes 197.01±740.78. There was no change in operation that can result in significant changes in the pumped volume over years, so operation of nuclear power plant should not be the cause of the extremely abundant individuals.

## Additional Information

**How to cite this article**: Chen, H. *et al.* Long-term monitoring dataset of fish assemblages impinged at nuclear power plants in northern Taiwan. *Sci. Data* 2:150071 doi: 10.1038/sdata.2015.71 (2015).

## Supplementary Material



## References

[d1] Dryad Chen H. 2015 http://dx.doi.org/10.5061/dryad.m777t

